# Possibilities for RNA Interference in Developing Hepatitis C Virus Therapeutics

**DOI:** 10.3390/v2081647

**Published:** 2010-08-06

**Authors:** Kristi L. Berger, Glenn Randall

**Affiliations:** Department of Microbiology, The University of Chicago, Chicago, IL 60637, USA; E-Mails: kberger@bsd.uchicago.edu

**Keywords:** RNA interference, hepatitis C virus, siRNA therapy

## Abstract

The discovery and characterization of the RNA interference (RNAi) pathway has been one of the most important scientific developments of the last 12 years. RNAi is a cellular pathway wherein small RNAs control the expression of genes by either degrading homologous RNAs or preventing the translation of RNAs with partial homology. It has impacted basic biology on two major fronts. The first is the discovery of microRNAs (miRNAs), which regulate almost every cellular process and are required for some viral infections, including hepatitis C virus (HCV). The second front is the use of small interfering RNAs (siRNAs) as the first robust tool for mammalian cellular genetics. This has led to the identification of hundreds of cellular genes that are important for HCV infection. There is now a major push to adapt RNAi technology to the clinic. In this review, we explore the impact of RNAi in understanding HCV biology, the progress in design of RNAi-based therapeutics for HCV, and remaining obstacles.

## 1. A brief history of RNA therapeutics

In the last 30 years, numerous novel roles for RNA in the regulation of gene expression have been discovered in addition to its traditional function as a conveyor of message from DNA to protein. Many of these RNA functions have been and will continue to be probed for therapeutic applications. The earliest therapeutic nucleic acids explored were antisense oligonucleotides, typically short (13–25 nucleotides (nts)) single-stranded DNA or RNA molecules. Hybridization of an antisense molecule with its target RNA results in either degradation by RNase H or steric hindrance of the mRNA splicing or translational machinery [reviewed in 1]. In 1998, the United States Food and Drug Administration approved the first antisense DNA drug, fomivirsen by Isis Pharmaceuticals, to treat cytomegalovirus retinitis in immuno-compromised patients [2], thus demonstrating that clinical application of therapeutic nucleic acids is an achievable goal.

Ribozymes and aptamers are RNA molecules identified in the 1980’s that were also tested for therapeutic potential. Hammerhead ribozymes, a class of catalytic RNAs (∼40 nts), are of particular interest because of their ability to recognize and cleave at specific RNA sequences [3,4]. Aptamers are highly structured RNAs (25–40 nts) with a high affinity for their protein ligand making them useful as possible drug inhibitors. For example, the transactivation response (TAR) aptamer encoded by HIV-1 binds the viral transactivator of transcription protein (Tat), and when over-expressed *in vitro*, TAR renders cells resistant to HIV-1 replication [5].

The newest and most promising addition to the field of RNA therapeutics arose from the discovery of the RNA interference (RNAi) pathway in 1998 by eventual Nobel Prize recipients Fire and Mello [6]. In *C. elegans*, Fire *et al.* noted injection of long, double stranded (ds) RNAs (∼300–1000 nts) complementary to a specified mRNA was accompanied by efficient elimination of the targeted transcript. This phenotype could not be reproduced in mammalian cells until Elbashir *et al.* provided a major breakthrough [7]. They showed that target mRNAs could be silenced by the transfection of cells with chemically synthesized siRNAs, which were designed to mimic the native siRNAs produced by RNAi in other systems [7]. It was subsequently shown that short hairpin RNAs (shRNAs), which mimic endogenous pre-miRNAs, could be expressed in cells and also yield effective target RNA silencing [8]. These studies have ushered in a new era of using siRNAs and shRNAs for mammalian genetics and therapeutic approaches. While RNAi-based therapeutics have not yet realized their considerable potential, much work is being done to advance these approaches into the clinic.

## 2. The RNAi pathway

**Figure 1.**
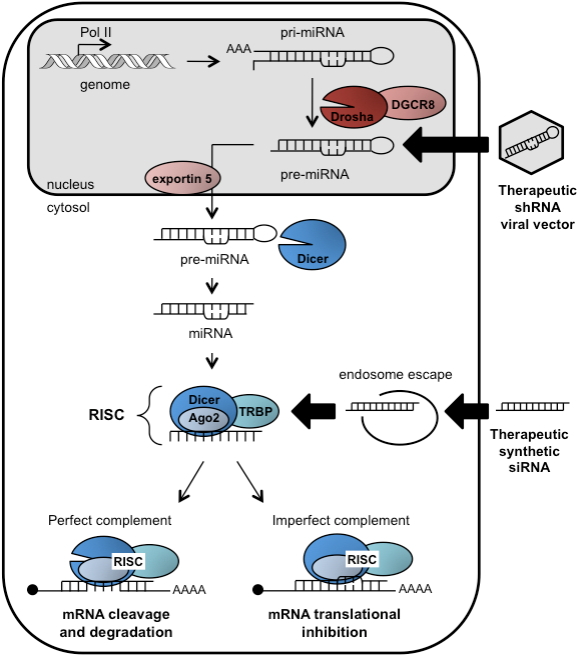
**Illustration of the endogenous RNAi pathway and therapeutic mimics.** Genome-encoded primary microRNAs (pri-miRNAs) are processed by Drosha into pre-miRNAs that are then exported from the nucleus. In the cytosol, Dicer cleaves pre-miRNAs into miRNAs and a single-stranded guide RNA is incorporated into RISC. Depending on sequence homology between the guide strand and the target, RISC either cleaves the mRNA or causes translational repression. The RNAi pathway can be activated by synthetic siRNAs or shRNAs to therapeutically treat metabolic disorders, cancers, or viral infections. shRNAs delivered by viral vectors mimic pre-miRNAs while siRNAs mimic the native miRNA duplexes and are incorporated into RISC. shRNAs must be exported from the nucleus and siRNAs delivered using liposome-based technologies must escape from endosomal compartments before being processed. Accessory proteins involved are DiGeorge syndrome critical region 8 (DGCR8), argonaute 2 (ago2), and HIV-transactivating response RNA-binding protein (TRBP).

RNAi is a conserved mechanism of post-transcriptional gene silencing (PTGS) identified in multiple organisms, from worms to plants to mammals. Much of the mechanism and key components of the RNAi pathway are now appreciated (Figure 1). In the cytoplasm, Dicer, a RNase III type enzyme, cleaves long dsRNAs into ∼21–25 nucleotide (nt) siRNAs, with 5′ phosphate groups and two nt 3′ overhangs [9,10]. The strand complementary to the target mRNA is called the guide strand while the other is called the passenger strand. Clues as to how Dicer recognizes its dsRNA substrate and how cleavage sites are chosen lie within its structure [11,12]. Dicer proteins usually contain a helicase/ATPase domain, two RNaseIII-like domains, a PAZ domain, and a dsRNA binding domain. The size of siRNAs produced are predicted by the distance between the PAZ and the RNaseIII domains, and the 3′ overhangs are a common feature produced by RNaseIII activity [11]. Several positively charged residues connecting these domains are thought to facilitate binding of RNA [11].

Dicer then delivers the siRNA to the RNA-induced silencing complex (RISC), in part consisting of Dicer, argonaute proteins, and HIV-transactivating response RNA-binding protein (TRBP) [13]. Argonaute 2 (Ago2) is the catalytic endonuclease at the heart of human RISC that binds the siRNA duplex, cleaves and removes the passenger strand, and thus leaves behind an intact single-stranded guide [14,15]. The strand with least complementarity at the 5′ end tends to serve as the guide [16]. Furthermore, TRBP binds to the more thermodynamically stable end of the siRNA and orients it with Ago2 in a way that helps identify the guide strand [17]. The degree of complementarity between the guide and target mRNA determines whether the mRNA is degraded (exact match) or translationally repressed due to steric hindrance (partial homology) [18]. In the case of exact homology, the RNase H domain of Ago2 is responsible for target mRNA cleavage [14].

Endogenous microRNAs (miRNAs) are the natural substrates for Dicer in mammals [reviewed in 18]. In the nucleus, RNA polymerase II (Pol II) transcribes genome-encoded primary miRNAs (pri-miRNAs) that can contain multiple hairpins [19]. A complex consisting of the RNase III enzyme Drosha and dsRNA binding protein DGCR8 cleaves the pri-mRNAs into 60–80 nt hairpins called pre-miRNAs [20]. Pre-miRNA hairpins are exported into the cytoplasm where they are recognized by Dicer and cleaved to generate the final miRNA duplex with characteristic two nt 3′ overhangs [20]. Novel Dicer-independent mechanisms for miRNA processing may also exist since it was reported that miR-451 cleavage relied only on Ago2 catalytic activity in zebrafish [21]. The majority of miRNA-derived guide strands have only partial target homology and are directed by RISC to the 3′ untranslated region (UTR) of mature mRNAs resulting in translational repression [22]. The “seed” region of the miRNA (nts 2–8 from 5′ end of the guide strand) is critical for recognition of target 3’UTRs [23]. More recently, it has been shown that central nucleotides can also guide substrate selection in some cases [24]. RNAi is now recognized as a critical regulator of virtually every cellular process.

RNAi has become a preferred method to perform highly specific genetic manipulations for functional studies in cell culture. RNAi studies have also greatly expanded the repertoire of possible drug targets to treat metabolic and genetic disorders, cancer, or viral infections. The underlying principle for RNAi-based therapy is to trigger PTGS using mimics of Dicer substrates (Figure 1): either chemically synthesized siRNAs or virally expressed shRNAs. Synthetic siRNAs can be delivered to Dicer in the cytosol by a variety of delivery approaches. shRNAs encoded in viral delivery vectors must be exported and processed by the endogenous miRNA pathway.

A study by Song *et al.* demonstrated the first *in vivo* siRNA application for disease therapy [25]. They showed that systemic delivery of siRNA duplexes targeting Fas, a mediator of hepatitis and fibrosis, resulted in hepatocyte-uptake and protected the treated mice from liver injury in a fulminant hepatitis mouse model. More *in vivo* studies for other diseases soon followed supporting the efficacy of siRNA-based therapies [reviewed in 26, 27]. An array of antiviral RNAi therapies have been corroborated in animal models, for example, influenza [28, 29], respiratory syncytial virus (RSV) [30, 31], parainfluenza virus [30], coxsackievirus B [32], SARS-associated coronavirus [33], West Nile virus [34], and herpes simplex virus (HSV-2) using a topical application [35].

## 3. RNAi and HCV

RNAi is considered an endogenous antiviral mechanism in plants and insects, but it is unlikely to be a robust antiviral pathway in mammalian somatic cells [36]. However, there is some evidence for limited antiviral activity of RNAi in mammalian cells. Minor processing of HCV RNAs, in addition to those of other viruses, into small RNAs during infection has been reported [37]. Interestingly, mammalian viruses that replicate in mosquito vectors, in which RNAi is a primary antiviral pathway, had extremely low levels of small RNAs identified in this study. This suggests that viruses that successfully infect insects may have evolved counter-strategies to RNAi. On the other hand, viruses that do not replicate in insect vectors, such as HCV, had higher levels of short viral RNAs, suggesting an absence of evolutionary pressure in mammals to express RNAi antagonists.

Contrary to the idea that RNAi is a mammalian antiviral mechanism, HCV in fact utilizes RNAi to support its infection. HCV replication and infectious virus production were inhibited when components of the RNAi pathway, including Dicer, were silenced by siRNAs [38]. This is likely related to a downstream processing effect: the loss of cellular miRNA-122 (miR-122). miR-122 is the most abundant miRNA in the liver [39] and studies suggest it plays a role in fatty acid and cholesterol metabolism [40]. During HCV infection, miR-122 binds to the 5’UTR of the HCV genome and regulates both HCV translation and replication by unknown mechanisms [41–44]. It has been shown that genotype 1b HCV replicon replication can be inhibited by sequestering miR-122 [41], and infectious J6/JFH-1 replication and virus production can be inhibited by anti-miR-122 RNAs [38]. A much more detailed description of HCV therapeutic approaches targeting miR-122 is described in this special issue of Viruses by Catherine Jopling [45]. Many other viruses, particularly the herpesviruses, co-opt the RNAi pathway by expressing viral miRNAs that modulate either cellular or viral gene expression [reviewed in 46].

Consistent with the lack of an HCV RNAi antagonist, many groups have shown that HCV replication is exquisitely sensitive to either chemically synthesized siRNAs or shRNA expression targeting HCV RNA. It was reported that siRNAs directly targeting HCV replicon RNAs, specifically at the 5′ untranslated region (UTR), core, NS3, NS4B, or NS5A, were effective at reducing viral replication and suggests siRNAs can be designed against most regions of the HCV genome [47–52]. The *in vitro* efficacy of siRNAs against fully infectious HCV has also been shown [38]. While the majority of HCV-siRNAs are complementary to the (+) strand, a reduction in both strands of the viral dsRNA replication intermediate has been observed [53]. It is plausible that targeting the (+) strand template indirectly leads to a decrease in synthesis of (−) strands. Although HCV mouse models are very limited, some groups have reported RNAi of HCV transgene expression in mice. One *in vivo* mouse study reported shRNAs specific to the HCV 5’UTR were effective at diminishing HCV internal ribosome entry site (IRES)-driven luciferase expression [54]. Another study using a similar method in mice reported silencing of a NS5B-luciferase transgene by NS5B-specific siRNAs [55].

Because dsRNAs can activate the interferon (IFN) pathway, it was necessary to address whether HCV-specific siRNAs could trigger IFN production. Kapadia *et al.* demonstrated that inhibition of viral replication by HCV RNAi *in vitro* was not associated with an upregulation of IFN-stimulated genes [47]. In fact, HCV siRNAs were better at reducing HCV RNA levels than high doses of IFN-α [47]. An analysis of combined RNAi and IFN treatment of HCV replicons in cell culture using lentivirus-delivered shRNAs has been performed. Results indicated that IFN-α did not interfere with gene silencing, and inhibition of HCV replication by HCV-specific shRNAs was enhanced by addition of IFN-α [56]. This underscores the possibility of combination therapies of siRNAs and IFN against HCV.

In addition to directly targeting the virus, one important use of RNAi in the HCV therapeutic arena that is likely to have success is the identification of novel drug targets by RNAi. In general, this approach uses siRNAs or shRNAs targeting cellular genes to identify host cofactors of HCV infection. Small molecule inhibitors can then be developed against the identified drug targets. Although host genes have traditionally been overlooked as antiviral targets, there is growing interest in the potential of host targets to minimize viral resistance and possibly for use as broad-spectrum antivirals. RNAi has been used on a small scale to verify that cofactors, such as CD81, PTB, La antigen, and VAP-A are important for HCV replication [57,58]. Many groups have recently published medium-to-high-throughput RNAi screens that identified hundreds of host factors utilized by HCV [38,59–64]. Identification of cellular proteins with enzymatic functions is ideal for development of novel, small molecule inhibitors and/or therapeutic siRNAs. Two such candidates are ubiquitin specific peptidase 18 (USP18) and phosphatidylinositol 4-kinase III alpha (PI4K-IIIα). siRNAs targeting USP18 have been shown to potentiate the ability of IFN-α to inhibit HCV replication and virus production [65]. PI4K-IIIα siRNAs dramatically reduce HCV replication suggesting this is a critical viral replication cofactor [61–64,66,67]. The identification and characterization of host cofactors utilized during HCV infection using RNAi technology is a significant advancement towards discovery of much needed new, therapeutic targets.

## 4. Advantages & pitfalls of RNAi therapeutics

Since HCV is a major public health burden and the current HCV therapy of IFN and ribavirin is successful in only half of treated patients [68], there are many alternative therapeutics under development. Most of these are traditional small molecule approaches inhibiting the function of viral proteins. RNAi-based antiviral strategies have a number of distinct advantages and disadvantages as compared with these therapies, which are discussed below. One of the primary advantages is that siRNAs are easy to design and synthesize, unlike small molecule inhibitors. Several guides and algorithms exist to aid in the choice of siRNA [69]. siRNAs must be experimentally tested for efficacy, but this is relatively trivial and typically amenable to high-throughput analysis. They also have a distinct target (RNA for siRNAs, *versus* protein for small molecule inhibitors), which yields promise for inclusion in multiple drug cocktails with distinct targets to limit viral escape. However, significant challenges remain with respect to RNAi therapy of HCV infection.

The first obstacle is the relative ease of viral resistance to a siRNA drug. HCV has a high mutation rate due to its error prone viral RNA polymerase, which leads to the emergence of drug-resistant viruses. In one study, siRNA-resistant HCV replicons emerged after four weeks of HCV-siRNA therapy with specific mutations in the target sequence [70]. In order to minimize the risk of drug resistance, multiple viral and/or cellular targets must be considered, as is the case with highly active antiretroviral therapy (HAART) for HIV. Delivery of multiple HCV siRNAs in parallel can be effective at limiting escape mutants [53,71]. Alternatively, siRNAs could be also combined with IFN treatment or viral protease and polymerase inhibitors. Another approach is to identify highly conserved regions of the HCV genome that are susceptible to siRNAs. These sequences should be less likely to mutate and provide resistance.

Another problem is the poor stability of siRNAs in serum which last only minutes due to the high concentration of nucleases present in blood [72]. However, naked siRNAs can be masked with chemical modifications for stability while maintaining sequence specificity [reviewed in 73]. A 2′-*O*-methyl addition is the most commonly used modification. The use of a phosphorothioate backbone linkage at the 3′ end or a 2′-fluoro sugar modification also offers protection against nuclease activity [74,75]. Furthermore, incorporation of fluoro-β-D-arabinonucleic acid or arabinonucleic acid can increase serum stability and potency [76,77].

siRNAs may deviate from their intended target and alter the expression from unintended mRNAs, which is termed an “off-target effect”. Off-target activity can occur when siRNAs target RISC to the 3’UTR of an unintended mRNA. This activity is mapped to the “seed” region described for miRNAs [78]. For miRNAs, translational repression occurs when there is exact pairing between the seed region (nts 2–8 of the guide strand) and its target 3’UTR, although a partial seed match can also trigger silencing given that there is sufficient base-pairing in other regions of the miRNA. This flexibility makes it difficult to predict potential off-target activity. For synthetic RNAs, RISC can integrate siRNAs into the miRNA pathway to produce translational repression of unintended mRNAs containing seed region homology. However, chemical modifications at nucleotide 2 of the seed region greatly reduce off-target effects. These include 2′-*O*-methyl, 2′-fluoro, 2’deoxy, or locked nucleic acids (LNAs) [79]. Additionally, a pool of siRNAs could be used wherein the concentration of an individual siRNA is reduced, thus minimizing off-target mRNA repression while enhancing specificity.

Unintended activation of the innate immune response is another complication. Some siRNAs have been shown to trigger innate immunity leading to cytokine and interferon production [80–82]. dsRNAs >30 nts activate protein kinase PKR and 2′,5′-oligoadenylate synthetase [83], but shorter siRNA duplexes minimize this activation. siRNAs can also be recognized by the mammalian toll-like receptors (TLRs) 3, 7, and 8 [81,84]. TLR3 is a dsRNA sensor, while TLR7 and 8 recognize ssRNA and dsRNA motifs. siRNAs targeting vascular endothelial growth factor (VEGF) or its receptor are being clinically tested for treatment of wet age-related macular degeneration (AMD). Interestingly, it was discovered that siRNAs from two different clinical trials interacted with and activated TLR3, thereby indirectly downregulating VEGF [85]. Thus, the siRNA activity is not sequence-specific and is unrelated to the RNAi mechanism. Some siRNAs can bind to TLR7 in a sequence-specific manner [81], but this can be remedied in some cases by adding a 2′-*O*-methyl modification [86]. Lastly, guanosine and uracil rich regions should be avoided in siRNA design as these may contribute to siRNA recognition by TLR7 and TLR8 [87,88].

An undesired off-target effect unique to viral delivery of shRNAs is the saturation of the RNAi machinery. Over-expression of shRNAs was found to saturate nuclear exportin 5, thereby inhibiting nuclear export and activity of cellular miRNAs [89]. Adeno-associated virus 8 (AAV8) is an attractive delivery vehicle for HCV therapies due to its improved liver transduction efficiency over other AAV vectors [90]. In a study by Grimm *et al.*, long-term over-expression of shRNAs from an AAV8 vector, delivered intravenously in mice, resulted in liver injury that was often fatal [91]. Again, this phenotype was pinpointed to competition between the shRNA and host miRNAs for limiting cellular factors. In theory, toxicity can be controlled by lowering the shRNA dosage, either by decreasing the amount of virus or the strength of shRNA promoter. Despite the disadvantages mentioned, several RNAi-based therapies have successfully made it to clinical trials. As with any potential therapeutic, issues of unintended consequences, such as toxicity, should be evaluated in standard pharmacokinetic and phase I safety studies.

## 5. siRNA drugs in clinical trials

siRNA drugs are promising treatments for many diseases [reviewed in 92–94]. The first generation of therapies was designed to treat wet AMD, the major cause of blindness in the United States in patients over the age of 55. The first highly anticipated siRNA therapy to advance to phase III clinical trials was intravitreal injection of bevasiranib (Opko Health), designed to target VEGF for treatment of wet AMD. While it showed promising activity in combination with an anti-VEGF antibody, ranibizumab (Genentech), the trial was predicted not to reach its therapeutic potential and was terminated in March 2009 [95]. In a collaboration between Merck-siRNA Therapeutics and Allergan, the AMD drug called AGN211745 (formerly sirna-027), which is a chemically modified siRNA targeting VEGF receptor 1, also held promise but did not pass phase II trials in 2009 [95]. Despite these two setbacks, PF-4523655 (formerly RTP801i-14) from Quark Pharmaceuticals and Pfizer is a siRNA currently in phase II trials for use in AMD and also diabetic macular degeneration.

Using a more advanced approach, CALAA-01 (Calando Pharmaceuticals) is a RNAi therapeutic candidate for treatment of solid tumors and consists of transferrin-coated cyclodextrin nanoparticles containing siRNAs that target the M2 subunit of ribonucleotide reductase (RRM2), a cancer target [96]. A recent report shows that systemic administration of CALAA-01, currently in phase 1 trials, specifically inhibits the intended mRNA transcript in humans [96], unlike the off-target effects reported for other clinical RNAi therapies [85]. This is the first evidence of a RNAi therapeutic in humans that indeed works by a RNAi mechanism. Dependent on upregulated transferrin receptors in cancer cells, this is also the first clinical siRNA therapy to utilize receptor-mediated delivery. Silence Therapeutics AG has developed a unique siRNA-lipoplex formulation to target protein kinase N3 (PKN3), an effector of the PI3-kinase pathway [97]. This drug (Atu027) is being explored in phase I trials for anti-angiogenesis therapy of advanced solid cancers. siRNA-mediated treatment to boost the immune response against metastatic melanoma is also in phase I (Duke University). For this study, patients are injected with autologous dendritic cells transfected with melanoma tumor antigen expression constructs and siRNAs targeting the immunoproteosome [98], presumably to enhance melanoma antigen presentation.

The first antiviral siRNA drug approved for clinical trials targets respiratory syncytial virus (RSV). RSV causes respiratory tract infections that are most severe in children. ALN-RSV01 (Alnylam Pharmacuetical) targets the RSV nucleocapsid and inhibits viral replication in the lung [99]. Findings from phase II trials, involving healthy adults experimentally inoculated with RSV, have now been published [99]. Both prevention and treatment modalities were incorporated into the trial design. In either modality, viral loads were diminished in patients treated with ALN-RSV01 as compared to placebo. However, the most significant antiviral effect was observed when it was administered prophylactically. A phase II trial testing efficacy of ALN-RSV01 in naturally infected lung transplant patients has been completed and now a phase IIb has begun [98]. Benitec, in collaboration with the City of Hope National Medical Center in California, published its phase I clinical trial for RNAi therapy of HIV-1 in June 2010 [100]. Autologous CD34+ cells from HIV patients with AIDS-related lymphoma were modified with a triple combination vector (rHIV7-shI-TAR-CCR5RZ) consisting of a shRNA targeting a tat/rev exon, a TAR RNA decoy, and a CCR5 targeting hammerhead ribozyme. This combination afforded long term suppression of HIV replication in a prior *in vitro* study [101], and the clinical results show it is feasible, well tolerated in patients, and has sustained expression up to 24 months [100].

Lastly, the safety of a therapeutic for HCV that targets miR-122 is being analyzed in phase I clinical trials. SPC3649 (Santaris Pharma) is a LNA-modified phosphorothioate oligonucleotide that was published to effectively silence miR-122 in African green monkeys [102] and produce prolonged suppression of viremia without HCV resistance in chronically infected chimpanzees [103] with tolerated reduction in cholesterol levels. Phase I clinical trials of SPC3649 began this year, with a competing anti-miR-122 antiviral developed by GlaxoSmithKline and Regulus Therapeutics scheduled for phase I trial in 2011 [104]. These clinical studies pave the way to designing effective RNAi strategies in patients.

## 6. The final frontier for RNAi therapeutics for HCV: effective siRNA delivery to the liver

The major challenge now is to progress from HCV *in vitro* “proof of concept” studies to *in vivo* therapeutic approaches using siRNAs or shRNAs. This has been especially challenging for HCV due to limited animal models. The greatest breakthrough for HCV RNA therapeutics thus far has not been siRNAs or shRNAs, but the anti-miR-122 drug SPC3649, which to date is the only successful treatment in a HCV animal model supportive of these approaches. By far the biggest hurdle in HCV siRNA therapy is the efficient delivery of siRNAs to the cytosol of infected hepatocytes. This entails maximizing both the uptake of siRNA by the liver and the escape of siRNAs from the endocytic compartments into the cytosol. The liver is an attractive organ for uptake of nucleic acids following systemic delivery as evidenced by several reports showing naked siRNAs are readily taken up by the liver in mice using hydrodynamic tail vein injection [25,55,105,106]. This delivery approach involves rapid, large volume injection and is not amenable to use in humans. The amounts of siRNAs required for a therapeutic effect against hepatitis B virus (HBV) using intravenous injection in an animal model were an order of magnitude above conceivable therapeutic doses in people [106]. Nevertheless, this method was important in first demonstrating siRNA efficacy in hepatocytes *in vivo*. There are multiple approaches being taken to improve uptake and delivery to the liver, which are outlined below.

One approach to improve specific organ delivery is conjugation of siRNAs to biologically relevant molecules. The strategy behind lipophilic modifications to siRNAs depends on binding to lipoproteins and internalization by low-density or high-density lipoprotein (LDL or HDL) receptors, which are highly expressed in the liver. Intravenous injection of cholesterol-conjugated siRNAs for apolipoprotein B (apoB) was effective at silencing transcripts in the liver and jejunum of mice, resulting in lowered cholesterol levels [107]. siRNAs conjugated to bile-salt derivatives or long chain fatty acids were also found to trigger RNAi in hepatocytes *in vivo* [108]. Vitamin E (α-tocopherol) is incorporated into lipoproteins after consumption, including LDLs and HDLs [109]. With this in mind, Nishina *et al.* showed that α-tocopherol-conjugated siRNAs (Toc-siRNAs) specific to apoB were effectively delivered and processed by Dicer in livers of mice without stimulating IFN production [110].

The negatively charged plasma membrane, circulating nucleases present in serum, and rapid clearance by the renal system impede uptake of siRNAs. One approach to overcome issues of membrane charge and nucleases is the incorporation of siRNAs into multilayered polyelectrolyte films (MPFs), which consist of alternating layers of polycations and polyanions wherein siRNAs are semi-protected from spontaneous release or diffusion until cell uptake [111]. Using this method, Dimitrova *et al.* showed effective and sustainable inhibition of HCV replication in cell culture using HCV siRNAs complexed within MPFs [111]. Another approach is the use of stable nucleic acid lipid particles (SNALPS) which contain siRNAs within a combination of cationic and fusogenic lipids coated with polyethelene glycol (PEG) [106]. SNALPS have been successfully used to deliver anti-HBV siRNAs to inhibit HBV replication in mouse hepatocytes and to deliver anti-apoB siRNAs in mice and monkeys [106,112]. Other types of cationic lipid-based vehicles have been developed based on a library of cationic lipid-like molecules, called lipidoids, that were generated by adding primary or secondary amines to alkyl-acrylates or alkyl-acrylamides [113]. The leading compound identified, 98N_12_-5(1), when combined with cholesterol and PEG lipid, effectively encapsulated and delivered siRNAs to mouse hepatocytes. Although, cationic liposomes have improved hepatic delivery, siRNA uptake is often observed in other organs. Also, liposome-siRNA based technologies face the problem of escaping the endosome once they are internalized. Endosomal release can be facilitated by incorporating fusogenic peptides and ligands or by using pH-sensitive nanocomplexes [reviewed in 114].

A unique technology called dynamic polyconjugates (DPCs) addresses issues of cell-specific targeting and endosomal escape. DPCs consist of the hepatocyte targeting ligand *N*-acetylgalactosamine conjugated to PEG (used as a shielding agent to prevent non-specific interactions of the nanoparticle) and to an endosomolytic polymer that is activated only in the acidic environment of the endosome. Using a single simple intravenous injection of anti-apoB siRNAs carried by DPCs, Rozema *et al.* reported specific reduction of *apoB* mRNA in the liver (hepatocytes specifically and not Kupffer cells) and not in the jejunum of mice [115], thus demonstrating the effectiveness of utilizing a targeting ligand.

Viral vectors are another means of triggering RNAi in hepatocytes. shRNAs are cloned into viral vectors under control of Pol II or Pol III promoters and typically packaged into lentivirus, adenovirus, or AAV particles. Viral encoded and delivered shRNAs have the benefit of long-term expression in dividing and non-dividing cells and incorporation of target ligands. To direct viral vectors to specific cells, target ligands can be incorporated into the viral particles or liver-specific RNA pol promoters used to drive shRNA expression. Viruses that have a natural tropism for certain tissues can be used, as in the case of hepatropic AAV8. However, as mentioned earlier, the downside of viral-encoded shRNAs is RNAi saturation and toxicity [91].

## 7. Conclusions and outlook

The identification of RNAi in mammalian cells is less than a decade old. However, the field has dramatically altered our fundamental understanding of gene regulation in almost every setting. It is clear that there is an enormous potential for RNAi-based therapies, which is as of yet unrealized. In the case of HCV, RNAi therapies will not be the next generation of antivirals to emerge from clinical trials. These almost certainly will be more classical protease and polymerase small molecule inhibitors that will be used in combination with IFN and ribavirin. Nevertheless, it is clear that additional therapeutic strategies involving novel targets will likely be required to treat patients who fail to respond to this combination therapy, which is proposed to be ∼15–25% of treated patients [116]. It is possible that RNAi therapeutics may be one of the next generations of drugs to help fill this niche.

The compelling strength of RNAi therapeutic approaches is that once the proper modifications are designed to minimize off-target effects, while maximizing effective delivery of siRNAs or shRNAs to the hepatic cytosol, they are universally applicable to any liver target. Only the siRNA nucleotide sequence needs to change in order to target any hepatotropic virus or liver gene target, e.g. to treat diseases with elevated LDL or cholesterol. Thus, if the issues with effective delivery are overcome, it will be trivial to apply this technology to many different hepatotropic diseases.
